# Sex differences in the relative contribution of social and clinical factors to the Health Utilities Index Mark 2 measure of health-related quality of life in older home care clients

**DOI:** 10.1186/1477-7525-7-80

**Published:** 2009-09-02

**Authors:** Colleen J Maxwell, Jian Kang, Jennifer D Walker, Jenny X Zhang, David B Hogan, David H Feeny, Walter P Wodchis

**Affiliations:** 1 Department of Community Health Sciences and Centre for Health & Policy Studies, University of Calgary, Calgary, Alberta, Canada; 2 Department of Medicine, University of Calgary, Calgary, Alberta, Canada; 3 Institute of Health Economics, Edmonton, Alberta, Canada; 4 Canadian Institute for Health Information, 495 Richmond Rd, Suite 600, Ottawa, Ontario, Canada; 5 Departments of Public Health Sciences and Economics, University of Alberta, Edmonton, Alberta, Canada; 6 The Center for Health Research, Kaiser Permanente Northwest, Portland, OR, USA; 7 Health Utilities Incorporated, Dundas, Ontario, Canada; 8 Health Policy Management and Evaluation, University of Toronto, Toronto, Ontario, Canada; 9 Institute for Clinical Evaluative Sciences and Toronto Rehabilitation Institute, Toronto, Ontario, Canada

## Abstract

**Background:**

The heterogeneity evident among home care clients highlights the need for greater understanding of the clinical and social determinants of multi-dimensional health-related quality of life (HRQL) indices and of potential sex-differences in these determinants. We examined the relative contribution of social and clinical factors to HRQL among older home care clients and explored whether any of the observed associations varied by sex.

**Methods:**

The Canadian-US sample included 514 clients. Self-reported HRQL was measured during in-home interviews (2002-04) using the Health Utilities Index Mark 2 (HUI2). Data on clients' sociodemographic, health and clinical characteristics were obtained with the Minimum Data Set for Home Care. The relative associations between clients' characteristics and HUI2 scores were examined using multivariable linear regression models.

**Results:**

Women had a significantly lower mean HUI2 score than men (0.48, 95%CI 0.46-0.50 vs. 0.52, 0.49-0.55). Clients with distressed caregivers and poor self-rated health exhibited significantly lower HRQL scores after adjustment for a comprehensive list of clinical conditions. Several other factors remained statistically significant (arthritis, psychiatric illness, bladder incontinence, urinary tract infection) or clinically important (reported loneliness, congestive heart failure, pressure ulcers) correlates of lower HUI2 scores in adjusted analyses. These associations generally did not vary significantly by sex.

**Conclusion:**

For females and males, HRQL scores were negatively associated with conditions predictive or indicative of disability and with markers of psychosocial stress. Despite sex differences in the prevalence of social and clinical factors likely to affect HRQL, few varied significantly by sex in their relative impact on HUI2 scores. Further exploration of differences in the relative importance of clinical and psychosocial well-being (e.g., loneliness) to HRQL among female and male clients may help guide the development of sex-specific strategies for risk screening and care management.

## Background

Health-related quality of life (HRQL) represents an important construct in understanding the health status and outcomes of older home care clients [1] and ultimately, the cost-effectiveness of community-based services and interventions [2]. The measurement of HRQL provides clinicians and researchers the opportunity of comparing diverse populations through a single measure that captures capacity in multiple domains of health that are relevant to older populations [3]. The heterogeneous nature of this frail population requires that program and policy makers clearly understand the determinants of multi-dimensional indices of health [4] and appreciate the potential for sex-differences in these determinants relevant for targeted or individualized care planning.

Research has demonstrated the negative impact of prevalent chronic conditions and multi-morbidity on the HRQL of older and vulnerable patient populations [5-11]. These studies have identified a number of conditions associated with notable decrements in older individuals' HRQL including, **a**rthritis, heart failure, stroke, chronic obstructive pulmonary disease (COPD), urinary incontinence and mental health disorders. Despite some suggestion of sex differences in the strength of the associations between selected chronic diseases and HRQL indices [11,12], current research has yet to provide a comprehensive analysis of sex differences specific to the older population, including those receiving community-based services.

Beyond clinical illness, few studies have explored the independent contribution of social factors to HRQL measures among older frail adults in the community. This is despite a well-developed theoretical and research basis on the importance of the social environment to several adverse health outcomes [13-15]. Early findings on the relevance of social relationships to functional impairment (an important dimension of HRQL) illustrate both the complex nature of these associations and the importance of differentiating among the structural and functional components of social ties [16,17]. A significant and independent association between "weak" social networks (e.g., having few friends and relatives, no confidant, little social integration) and lower scores on the mental [18] and physical [4] components of HRQL has been reported for women participating in the US Nurses' Health Study. A prospective study of this cohort also showed a *lower *risk for decline in mental health among older women living alone (vs. with a spouse), possibly reflecting their increased levels of social integration and engagement with friends, extended family and social groups [19,20]. Recent cross-sectional population-based studies of community-residing older adults have found significant associations between infrequent contact with family and friends and lower scores on selected components of the SF-12 [21] and SF-36 measures of HRQL [22]. Although these findings persisted after adjusting for aggregate measures of comorbidity, the relative contribution and potential confounding effects of individual health conditions were not considered.

The possibility of sex differences has also been raised with regard to the associations between social relations and HRQL in older adults. The underlying rationale arises from observed sex differences in social vulnerability and in the associated risks for adverse health outcomes, including mortality and functional decline [20,23,24]. Findings suggest worse outcomes among men who live alone (or are socially isolated) compared with women. Conversely, other studies examining the relationship between social support/integration and single-item (self-reported health) and multi-dimensional HRQL measures have found little evidence for effect modification by sex [22,25].

The objectives of the current study were two-fold, first to examine, in a comprehensive manner, the relative contributions of social *and *clinical factors to the HUI2 measure of health-related quality of life (HRQL) among older adults receiving home care services; and, second, to explore whether any of the observed associations between these client factors and HRQL varied by sex. Our aims and analytical approach were guided by well-developed conceptual frameworks [13,15] illustrating the relevance of a diverse range of sociodemographic, clinical and psychosocial factors to health and well-being in vulnerable populations.

## Methods

### Sample

Data were derived from comprehensive in-home assessments conducted with older home care clients in Calgary, Alberta, Canada and Wayne County, Michigan, USA between February 2002 and April 2004 [26,27]. Clients able to communicate (in English) and provide informed consent, as determined by nurse assessors and home care staff, were eligible for participation. In Calgary, the sample included participants aged 65 and older in a Comprehensive Community Care (C3) program and a control group of home care clients matched to C3 clients by residence and clinical status [27]. Of the 168 clients enrolled in the C3 program during the initial two years of its operation, 114 provided informed consent for study participation. Of the 182 control home care clients invited to participate during the study period, 150 provided informed consent and were assessed. The Wayne County sample included both new and existing older home care clients. From the latter sample, 35 clients were not eligible for the study and four refused to participate. The final study sample consisted of 514 participants, 264 from Calgary and 250 from Michigan. The study received ethics approval from the Conjoint Health Research Ethics Board, University of Calgary.

### Data Collection

Clients were administered a standardized multi-dimensional assessment instrument, the Minimum Data Set for Home Care (MDS-HC) [28], and the Health Utilities Index Mark 2 (HUI2) 40-item interviewer-administered, self-assessed ''one-week'' health status assessment questionnaire [29]. The study home care nurses/case-coordinators were trained by the principal investigators and administered the HUI2 survey via a face-to-face interview. In both settings, the MDS-HC had been adopted as the primary assessment and care planning tool in the routine clinical management of clients. The MDS-HC provides a comprehensive assessment of clients' sociodemographic, physical and cognitive status, health conditions, mental and social well-being, behaviour, formal and informal service use, and use of prescription and over-the-counter medications. The time frame for most items is the seven days preceding the assessment date. Assessors are trained to consider all relevant sources of information (to ensure completeness and accuracy) including the client, direct observation, formal and informal (family/friends) caregivers and medical records. Preference is given to clients' self-reported responses and information sources deemed to be most accurate and reliable for specific assessment items (e.g., medical records for clinical items). The reliability and validity of the MDS-HC instrument for both clinical and research purposes have previously been established [28,30,31].

### Measures

The HUI2 is a generic multi-attribute utility index measure that has been extensively used in clinical studies and population health surveys [32]. Evidence in support of the reliability, validity and responsiveness of the HUI system exists for diverse patient populations [33,34], including older adults receiving community-based care [26,27]. All responses were self-reported by clients and no proxy responses were used. The HUI2 consists of seven attributes (domains): sensation (vision, hearing and speech), mobility, emotion, cognition, self-care, pain and fertility (not assessed here). We specifically examined HUI2 rather than HUI3 scores because of the inclusion of the self-care domain (not addressed by the HUI3), a particularly important contribution to HRQL in older home care clients.

Two steps are required to derive overall HUI2 scores. The first is to classify clients according to their self-reported functional capacity in each attribute, accomplished by mapping responses from the survey questionnaire to the HUI2 classification system. There are four or five levels per attribute ranging from normal/full function to severe disability. The second step is to associate general population preference-weights for the client's health state. As done previously [34], we applied the standard published Canadian preference weights [35] in our analyses. The overall HUI2 ranges from -0.02, allowing for states worse than dead (scored at 0.0), to 1.0, the best possible health one could expect to achieve. A differential of 0.03 in the overall HUI2 score indicates a clinically important difference (i.e., one that has meaning to an individual on a daily basis and may affect their medical care) [29]. Single-attribute utility scores are also available and range from 0.00 (the most severely impaired level) to 1.00 (no impairment). Differences of 0.05 or more in single-attribute utility scores are generally regarded as clinically important [29].

For incomplete observations due to missing responses to individual HUI survey questions (less than 5% of sample), we imputed missing (categorical) HUI questionnaire items with the modal response values. We included the imputed data in all analyses. Alternative imputation techniques did not affect our results.

The MDS-HC items examined as potential correlates of HUI2 overall scores were selected on the basis of previous literature and relevant conceptual models [14,15]. The items included measures of clients' demographic characteristics (age, sex); social relationships - including structural (marital status) and functional (feelings of loneliness and caregiver distress) elements; perceived self-rated health; health conditions (bladder incontinence, pressure ulcers, urinary tract infections in the past 30 days); and, type and number of chronic disease diagnoses (including, cerebrovascular disease, congestive heart failure, coronary artery disease, hypertension, peripheral vascular disease, multiple sclerosis, parkinsonism, arthritis, osteoporosis, any psychiatric illness, cancer, diabetes, chronic obstructive pulmonary disease, renal failure and thyroid disease). The disease diagnoses section of the MDS-HC includes a list of conditions to be assessed as present where: (i) indicated by a physician and/or the medical record (and noted to affect the client's status); and/or (ii) treatment or monitoring by a home care professional is required; or (iii) the disease was the reason for a hospitalization in the past 90 days. Caregiver stress or burden was coded as present if a positive response was coded for any of the following three MDS-HC items: 1) the client's caregiver is unable to continue in caring activities (e.g., due to decline in her/his own health); 2) the client's caregiver is not satisfied with the support received from family and friends; and/or 3) the client's caregiver expresses feelings of distress, anger or depression. Clients' perceived or self-rated health (poor vs. good) was based on the single MDS-HC question item, "Client feels she/he has poor health when asked." Despite some conceptual overlap with the HUI2 measure, we included self-rated health among our correlates given its observed potential to capture unique dimensions of health and well-being of prognostic significance [36].

For most correlates, missing data accounted for less than one percent of the total sample. The one exception was the item on pressure ulcers where greater than five percent of the total sample had missing data. Because of the possibility that clients with missing data for this variable were in some manner unique, we included missing values for the pressure ulcer item as a separate dummy variable in the regression analyses. Other variables of potential interest (e.g., education, living arrangements) were not assessed in a comprehensive manner in both home care samples and thus were not examined in this study.

### Analysis

Descriptive statistics were calculated and comparisons between men and women in clients' demographic, social, health and clinical characteristics were evaluated using *t*-tests for continuous variables and *chi-square *tests for categorical variables. Attribute-specific scores for women and men were also compared for each of the six health attributes included in the HUI2 using nonparametric tests for data not normally distributed. Unadjusted and age/sex-adjusted linear regression models were used to examine initial differences in mean HUI2 scores across each client variable. The relative significance of selected demographic, social, health and clinical factors to overall HUI2 scores was examined for the total sample using multivariable linear regression models. Factors associated with HUI2 scores in the bivariate analyses (either statistically at p < 0.05 or clinically as illustrated by a meaningful difference in mean scores of ≥ 0.03) were included in the multivariable analysis. Sex-specific multivariable models were conducted to examine the relative importance of clients' characteristics to HRQL separately for women and men. Any observed sex differences were further examined with interaction terms incorporated in the full model for the total sample. Because the analyses were viewed as being exploratory, no adjustment was made for multiple testing. Twenty-five clients were excluded from the multivariable analyses because of missing values for selected covariates resulting in a final sample size of 489 clients. All analyses were performed using SAS, Version 9.1.

## Results

### Sample characteristics (Table 1)

**Table 1:** Distribution^a ^of Demographic, Social, Health and Clinical Characteristics of Older Home Care Clients assessed during 2002-2004

	**Total** **(n = 514)**	**Female Clients** **(n = 372)**	**Male Clients** **(n = 142)**	**p value^b^**
**Age mean (SD)**	80.5 (8.4)	81.4 (7.9)	78.0 (9.3)	0.0002
**< 75**	137 (26.7)	85 (22.9)	52 (36.6)	0.0018
**75-84**	222 (43.2)	162 (43.6)	60 (42.3)	
**85+**	155 (30.2)	125 (33.6)	30 (21.1)	
**Marital Status**				< 0.0001
**Married**	158 (30.7)	70 (18.8)	88 (62.0)	
**Widowed**	279 (54.3)	244 (65.6)	35 (24.7)	
**Other**	77 (15.0)	58 (15.6)	19 (13.4)	
**Caregiver Stress**				0.0890
**no indicators**	423 (83.3)	312 (85.0)	111 (78.7)	
**1+ indicator(s)**	85 (16.7)	55 (15.0)	30 (21.3)	
**Reports Feels Lonely**				0.0197
**no**	390 (77.1)	270 (74.2)	120 (84.5)	
**yes**	116 (22.9)	94 (25.8)	22 (15.5)	
**Self-Rated Health**				0.0118
**good/excellent**	349 (69.0)	240 (65.8)	109 (77.3)	
**poor**	157 (31.0)	125 (34.3)	32 (22.7)	
**Arthritis**				< 0.0001
**no**	226 (44.0)	143 (38.4)	83 (58.5)	
**yes**	288 (56.0)	229 (61.6)	59 (41.6)	
**Congestive Heart Failure**				0.1984
**no**	386 (75.1)	285 (76.6)	101 (71.1)	
**yes**	128 (24.9)	87 (23.4)	41 (28.9)	
**Peripheral Vascular Disease**				0.1185
**no**	440 (85.6)	324 (87.1)	116 (81.7)	
**yes**	74 (14.4)	48 (12.9)	26 (18.3)	
**Hypertension**				0.1639
**no**	228 (44.4)	158 (42.5)	70 (49.3)	
**yes**	286 (55.6)	214 (57.5)	72 (50.7)	
**Emphysema/COPD**				0.2158
**no**	399 (77.6)	294 (79.0)	105 (73.9)	
**yes**	115 (22.4)	78 (21.0)	37 (26.1)	
**Osteoporosis**				< 0.0001
**no**	364 (70.8)	240 (64.5)	124 (87.3)	
**yes**	150 (29.2)	132 (35.5)	18 (12.7)	
**Psychiatric Diagnosis**				0.9416
**no**	368 (71.6)	266 (71.5)	102 (71.8)	
**yes**	146 (28.4)	106 (28.5)	40 (28.2)	
**No. of Chronic Conditions**				0.9036
**0-2**	127 (24.7)	90 (24.2)	37 (26.1)	
**3-5**	249 (48.4)	181 (48.7)	68 (47.9)	
**6+**	138 (26.9)	101 (27.2)	37 (26.1)	
**Bladder Incontinence**				< 0.0001
**no**	305 (61.0)	201 (55.7)	104 (74.8)	
**yes**	195 (39.0)	160 (44.3)	35 (25.2)	
**Pressure Ulcer**				0.0177
**no**	454 (88.3)	328 (88.2)	126 (88.7)	
**yes (any stage 1-4)**	20 (3.9)	10 (2.7)	10 (7.0)	
**missing value**	40 (7.8)	34 (9.1)	6 (4.2)	
**Urinary Tract Infection**				0.7902
**no**	496 (96.5)	358 (96.2)	138 (97.2)	
**yes**	18 (3.5)	14 (3.8)	4 (2.8)	

^a ^Frequency (%) unless otherwise indicated; ^b^test for sex difference.

The mean age of our sample was 80.5 (sd 8.4) years and 72 percent (n = 372) were women. Female clients were significantly older than male clients and more likely to be widowed, to report feelings of loneliness and poor self-rated health, and to have arthritis, osteoporosis, and bladder incontinence. Male clients were significantly more likely to be assessed as having pressure ulcers, whereas females were more likely to have missing data for this variable. Males were also slightly more likely to have distressed family caregivers. The distribution of other prevalent chronic conditions and overall number of chronic disease diagnoses did not vary significantly between female and male clients. The median number of chronic diseases was 4 (interquartile range 3-6) with a range of 0-13 conditions in the total sample.

### HUI2 Mean Overall and Single Attribute Scores (Table 2)

**Table 2:** HUI2 Mean (95%CI) Overall and Single Attribute Scores among the Total Sample of Older Home Care Clients and by Client Sex

	**Overall^a^**	**Cognition**	**Emotion**	**Sensation**	**Pain^b^**	**Mobility^c^**	**Self-care**
**Total** (n = 514)	0.49 (0.48-0.51)	0.89 (0.88-0.90)	0.85 (0.83-0.87)	0.79 (0.77-0.81)	0.77 (0.75-0.79)	0.57 (0.55-0.59)	0.43 (0.39-0.47)
**Females** (n = 372)	0.48 (0.46-0.50)	0.89 (0.88-0.91)	0.85 (0.82-0.87)	0.79 (0.78-0.81)	0.75 (0.72-0.78)	0.56 (0.54-0.58)	0.43 (0.38-0.48)
**Males** (n = 142)	0.52 (0.49-0.55)	0.89 (0.87-0.91)	0.86 (0.82-0.90)	0.78 (0.75-0.82)	0.82 (0.77-0.86)	0.60 (0.56-0.64)	0.44 (0.36-0.53)

^a^Significantly lower value for female vs. male clients, ^a^p = 0.0367; ^b^p = 0.0051.

^c^Clinically important difference for female vs. male clients, p = 0.0976

In this predominantly older female home care sample, the overall mean HUI2 score was 0.49 (95% CI 0.48-0.51). Women had significantly lower mean HUI2 scores than men (0.48, 95% CI 0.46-0.50 vs. 0.52, 95% CI 0.49-0.55). The relative impairment among HUI2 attributes showed decreasing scores in the following order: cognition, emotion, sensation, pain, mobility and self-care. This pattern was consistent for females and males with the exception of the pain attribute where the mean score was significantly lower for women (0.75, 95% CI 0.72-0.78) than men (0.82, 95% CI 0.77-0.86). The mean score for the mobility attribute was also lower among female compared with male clients (0.56, 95% CI 0.54-0.58 vs. 0.60, 95% CI 0.56-0.64).

### Unadjusted and Adjusted Differences in Mean HUI2 Scores across Client Characteristics (Table 3)

**Table 3:** Mean (SD) HUI2 Scores and Adjusted Differences by Demographic, Social, Health and Clinical Characteristics of Older Home Care Clients

	**N (%)**	**HUI2 Mean^a ^(SD)**		**Regression Coefficient^b^(SE)**	
**Overall**	489^c^	0.49 (0.18)			
**Sex**					
**Female**	351 (71.8)	0.48 (0.18)		Ref	
**Male**	138 (28.2)	0.52 (0.19)	p = 0.0420	0.01 (0.02)	p = 0.4369
**Age Group**					
**< 75**	128 (26.2)	0.50 (0.20)		Ref	
**75-84**	212 (43.4)	0.48 (0.19)	p = 0.4136	-0.03 (0.02)	p = 0.2044
**85+**	149 (30.4)	0.51 (0.16)	p = 0.6418	-0.01 (0.02)	p = 0.7953
**Marital Status**					
**married**	151 (30.9)	0.48 (0.20)	p = 0.9610	----	
**widowed**	266 (54.4)	0.50 (0.18)	p = 0.5595	----	
**other**	72 (14.7)	0.48 (0.18)		----	
**Caregiver Stress**					
**no indicators**	409 (83.6)	0.51 (0.18)		Ref	
**1+ indicator(s)**	80 (16.4)	0.40 (0.17)	p < 0.0001	-0.09 (0.02)	p < 0.0001
**Reports Feels Lonely**					
**no**	378 (77.3)	0.50 (0.19)		Ref	
**yes**	111 (22.7)	0.46 (0.18)	p = 0.0783	-0.03 (0.02)	p = 0.1655
**Self-Rated Health**					
**good/excellent**	339 (69.3)	0.52 (0.18)		Ref	
**poor**	150 (30.7)	0.42 (0.18)	p < 0.0001	-0.07 (0.02)	p = 0.0002
**Arthritis**					
**no**	214 (43.8)	0.53 (0.18)		Ref	
**yes**	275 (56.2)	0.46 (0.18)	p < 0.0001	-0.04 (0.02)	p = 0.0082
**Congestive Heart Failure**					
**no**	365 (74.6)	0.50 (0.19)		Ref	
**yes**	124 (25.4)	0.46 (0.17)	p = 0.0486	-0.03 (0.02)	p = 0.0622
**Peripheral Vascular Disease**					
**no**	416 (85.1)	0.50 (0.18)		Ref	
**yes**	73 (14.9)	0.46 (0.19)	p = 0.1274	-0.02 (0.02)	p = 0.4618
**Hypertension**					
**no**	218 (44.6)	0.50 (0.19)		----	
**yes**	271 (55.4)	0.48 (0.18)	p = 0.1505	----	
**Emphysema/COPD**					
**no**	378 (77.3)	0.50 (0.18)		Ref	
**yes**	111 (22.7)	0.47 (0.19)	p = 0.1485	0.004 (0.02)	p = 0.8301
**Osteoporosis**					
**no**	348 (71.2)	0.50 (0.19)		Ref	
**yes**	141 (28.8)	0.47 (0.17)	p = 0.0517	-0.01 (0.02)	p = 0.6749
**Psychiatric Diagnosis**					
**no**	351 (71.8)	0.51 (0.18)		Ref	
**yes**	138 (28.2)	0.44 (0.18)	p < 0.0001	-0.04 (0.02)	p = 0.0399
**Bladder Incontinence**					
**no**	298 (60.9)	0.52 (0.19)		Ref	
**yes**	191 (39.1)	0.45 (0.17)	p < 0.0001	-0.05 (0.02)	p = 0.0064
**Pressure Ulcer**					
**no**	440 (90.0)	0.49 (0.18)		Ref	
**yes (any stage 1-4)**	20 (4.1)	0.43 (0.10)	p = 0.1634	-0.03 (0.04)	p = 0.4338
**missing value**	29 (5.9)	0.48 (0.22)	p = 0.7278	-0.08 (0.04)	p = 0.0142
**Urinary Tract Infection**					
**no**	472 (96.5)	0.49 (0.19)		Ref	
**yes**	17 (3.5)	0.40 (0.15)	p = 0.0325	-0.09 (0.04)	p = 0.0299

^a ^Bivariate results from simple (unadjusted) linear regression models, comparisons with reference group.

^b ^Obtained from multivariable linear regression model, adjusting for all variables listed in table (R^2 ^0.18).

^c ^25 clients were excluded due to missing values for covariates.

The bivariate analyses revealed significantly lower mean HUI2 scores among clients with distressed caregivers, poor self-rated health, arthritis, congestive heart failure, any psychiatric diagnosis, bladder incontinence, and urinary tract infections. Clinically important differences (i.e., difference of ≥ 0.03 in HUI2 scores) were observed for clients reporting feeling lonely and those with osteoporosis, pressure ulcers, peripheral vascular disease, and emphysema/COPD. Mean overall HUI2 scores did not vary significantly by clients' age or marital status or for any of the other chronic diseases examined (including hypertension, cerebrovascular disease, coronary artery disease, multiple sclerosis, parkinsonism, cancer, diabetes, renal failure and thyroid disease).

As illustrated by the multivariable linear regression results, the strongest associations with overall HUI2 scores were observed for high caregiver stress and poor self-rated health. Several other factors remained statistically significant (arthritis, psychiatric diagnosis, bladder incontinence, urinary tract infection) or clinically important (client feels lonely, congestive heart failure, pressure ulcers) correlates of lower HUI2 scores in the adjusted analyses (model R^2 ^of 0.18). Although it is possible to calculate indices of clients' functional (i.e. activities of daily living [ADL]), cognitive and emotional status from MDS-HC items, we chose not to present the adjusted models including these scales because of interpretation difficulties due to collinearity between these measures (and other correlates of interest) and the single attributes comprising the HUI2 [26]. Adjusting for these additional measures did not substantially alter our original model estimates observed for clients' characteristics or conclusions. There was no significant difference in HUI2 scores among female and male clients after adjusting for selected health and clinical factors.

### Adjusted Differences in Mean HUI2 Scores across Client Characteristics, by Sex (Table 4)

**Table 4:** Adjusted Differences in HUI2 Scores by Demographic, Social, Health and Clinical Characteristics of Older Female and Male Home Care Clients

	**Regression Coeff.^a ^(SE) *Females***		**Regression Coeff.^a ^(SE) *Males***	
**Age Group**				
**< 75**	Ref		Ref	
**75-84**	-0.03 (0.02)	p = 0.2009	-0.01 (0.04)	p = 0.7350
**85+**	-0.004 (0.03)	p = 0.8771	-0.005 (0.05)	p = 0.9168
**Caregiver Stress**				
**no indicators**	Ref		Ref	
**1+ indicator(s)**	-0.08 (0.03)	p = 0.0019	-0.11 (0.04)	p = 0.0081
**Reports Feels Lonely**				
**no**	Ref		Ref	
**yes**	-0.02 (0.02)	p = 0.3289	-0.04 (0.04)	p = 0.3205
**Self-Rated Health**				
**good/excellent**	Ref		Ref	
**poor**	-0.06 (0.02)	p = 0.0056	-0.08 (0.04)	p = 0.0478
**Arthritis**				
**no**	Ref		Ref	
**yes**	-0.05 (0.02)	p = 0.0093	-0.03 (0.03)	p = 0.3807
**Congestive Heart Failure**				
**no**	Ref		Ref	
**yes**	-0.03 (0.02)	p = 0.1634	-0.03 (0.04)	p = 0.3398
**Peripheral Vascular Disease**				
**no**	Ref		Ref	
**yes**	-0.03 (0.03)	p = 0.3191	0.01 (0.04)	p = 0.7597
**Emphysema/COPD**				
**no**	Ref		Ref	
**yes**	0.02 (0.02)	p = 0.3609	-0.008 (0.04)	p = 0.8339
**Osteoporosis**				
**no**	Ref		Ref	
**yes**	-0.003 (0.02)	p = 0.8880	-0.02 (0.05)	p = 0.6497
**Psychiatric Diagnosis**				
**no**	Ref		Ref	
**yes**	-0.05 (0.02)	p = 0.0265	-0.03 (0.04)	p = 0.3819
**Bladder Incontinence**				
**no**	Ref		Ref	
**yes**	-0.05 (0.02)	p = 0.0085	-0.04 (0.04)	p = 0.3619
**Pressure Ulcer**				
**no**	Ref		Ref	
**yes (any stage 1-4)**	-0.05 (0.06)	p = 0.4011	-0.001 (0.06)	p = 0.9813
**missing value**	-0.06 (0.04)	p = 0.1078	-0.17 (0.09)	p = 0.0508
**Urinary Tract Infection**				
**No**	Ref		Ref	
**Yes**	-0.05 (0.05)	p = 0.3461	-0.23 (0.10)	p = 0.0168

	**n = 351, R^2 ^0.17**		**n = 138; R^2 ^0.22**	

^a ^Obtained from multivariable linear regression model, adjusting for all variables listed in table.

Among both female and male clients, high caregiver stress and poor self-rated health were significantly associated with lower overall HUI2 scores. Statistically lower scores were also observed among female clients with arthritis, any psychiatric illness and bladder incontinence. These three conditions were also associated with clinically important lower overall HUI2 scores among male clients. The presence of a urinary tract infection was associated with significantly lower overall HUI2 scores in males and with clinically important lower scores in females. Congestive heart failure was associated with lower HUI2 scores in both female and male clients. Female clients assessed as having a pressure ulcer (any stage) displayed clinically important lower HUI2 scores. Some caution is warranted in the interpretation of the sex-differences observed for pressure ulcers and urinary tract infections given the relative low occurrence of these conditions and missing data (for pressure ulcers). A clinically important difference in HUI2 scores was observed for male (but not female) clients reporting feelings of loneliness. None of the two-way interaction terms with sex were found to be statistically significant at p < 0.05 in the full model.

## Discussion

Older home care clients often show considerable variability in physical, cognitive and social indices of vulnerability. The relative frailty of our client sample is reflected by their significantly lower overall mean HUI2 scores (0.48 for female and 0.52 for male clients) compared with published age-specific HUI2 norms (e.g., 0.82 and 0.84 for female and male US adults aged 75-89, respectively) [32]. The lower scores largely reflect impairment in the self-care, mobility and pain attributes of the HUI2 measure consistent with the observed importance of disability-related conditions (e.g., arthritis, urinary incontinence and mental health disorders) to the HRQL of older adults [6-9]. Few studies have comprehensively examined the relative contribution of selected health *and *social factors to multi-dimensional HRQL indices such as the HUI2. Even less consideration has been paid to potential sex-differences in the relative importance of selected associations. In addition to highlighting the relative importance of the above disability-related conditions we found statistically significant and clinically important decrements in clients’ HRQL associated with poor selfrated health, urinary infections and caregiver distress (a *possible* indicator of a weak or unsettled social environment). Clients’ HUI2 scores varied little with age, marital status, or the presence of several common disease diagnoses. HUI2 scores were clinically reduced for those with self-reported loneliness, congestive heart failure and pressure ulcers. Generally, the observed associations were not significantly modified by sex; however, the magnitude of score differences associated with selected factors (e.g., arthritis, psychiatric illness, urinary tract infection) did vary between females and males. 

The relatively weak associations between individual disease diagnoses and clients' HRQL have also been reported by others [6,7,37] and may be expected for relatively stable and effectively treated conditions like hypertension. Conversely, others have noted a significant impact of osteoporosis [10], respiratory disorders [9] and stroke [11] on the HRQL of older populations. This inconsistency may reflect variations across studies in the measure of HRQL examined, in disease severity and treatment [10] and in the degree of co-occurrence among conditions assessed [6]. As with others [9,37], we examined the independent association between each separate disease diagnosis and overall HRQL scores. Further work is needed to examine the possible synergistic effects among co-morbid conditions in relation to HRQL among older populations [5]. This research should include sex-stratified analyses given that several relevant conditions (e.g., urinary incontinence, musculoskeletal and psychiatric disorders) are more prevalent among older women and may exhibit a relatively greater impact on the HRQL of women than men [12,38].

In interpreting the relatively strong impact of poor self-rated health and caregiver distress on the HRQL of female and male clients it is important to acknowledge that these factors may serve as proxy measures for poorer clinical and functional status. Yet, both remained significantly associated with lower HUI2 scores after adjusting for clients' clinical and functional status including additional MDS-HC measures of ADL and cognitive impairment. Consequently, a broader interpretation might suggest that both factors provide unique information about clients' psychosocial well-being (including level of psychological distress) relevant to their overall HRQL [36,39]. Additional longitudinal research is required to further delineate the nature and direction of these associations.

Widowhood and reported loneliness, although significantly more common among women, were not significantly associated with lower HRQL. Self-reported loneliness did, however, show a clinically important association with lower HUI2 scores among male clients. Others have reported weak to no associations between both marital status and living arrangements (i.e., living alone) and HRQL in older populations [8,21,22]. Arguably, the relevance of these social factors may vary depending on the dimension of HRQL and nature and function of social ties examined [16,17,40]. For example, older persons living alone have been observed to have lower scores on the mental components of HRQL, whereas those living with family have been found to have lower scores on the physical dimensions [41] reflecting the importance of families in providing care with ADL and positive emotional support [14]. Others have demonstrated the relative importance of friendship ties and level of social integration to the HRQL of older populations [21,22]. It remains unclear whether the associations found for social support and integration and HRQL measures vary by sex. Some investigators have failed to observe any effect modification by sex [22,25] whereas others have noted increased risks for poor health outcomes among men (but not women) who live alone or are socially isolated [14,24]. Older women living alone may be better positioned than men to call upon a more extensive social network of friends and community organizations for support and care [19,20]. Thus, despite being more common among older women, living alone may be more closely tied with loneliness (and possibly, poorer outcomes) among older men. Given the complexity of the relationships between social ties and HRQL and potential for sex differences in the underlying biological pathways [42] further research in this area is warranted.

Our cross-sectional study precludes any clear discussion of the causal nature of selected observed associations (e.g., between clients' social characteristics, caregiver distress, and perceived well-being and HRQL). The absence of longitudinal data also limits our ability to detect and comment on relevant changes in clients' HRQL precipitated by expected fluctuations in their clinical and health conditions [43]. Consistent with others [6,7], our multivariable model accounted for a relatively small proportion of explained variance in overall HUI2 scores illustrating the need to consider a broader range and/or more detailed measures of psychosocial, health and lifestyle factors potentially relevant to the HRQL of older populations. Comprehensive data regarding clients' education, income, rural/urban residence and lifestyle practices were not available for our sample and thus could not be examined in our analyses. Although relevant to HRQL measures in the US [44] and elsewhere [21,22], income and education have generally not been found to be significant predictors of HRQL for older adults in the Canadian context [44]. There is, however, increasing evidence for the importance of lifestyle factors (e.g., low physical activity, smoking) in accounting for lower HRQL in older populations [44-46]. Further work is required to explore possible sex differences in the prevalence and relative impact of these lifestyle factors to the HRQL of older adults given that such factors may be amenable to targeted interventions.

With our relatively select and small sample, some caution is warranted in generalizing our findings to other older populations and care settings including those in rural areas, in long-term care institutions and with moderate to severe cognitive impairment. Our relatively small number of male clients may also explain the relative absence of significant sex differences. As with other commonly used HRQL measures the HUI2 is particularly sensitive to physical impairment [1]. Also, in using the HUI2 (a generic indirect multi-attribute utility index measure of HRQL) our study did not directly assess or compare the preferences of female and male clients for various health states. Thus, different conceptual and methodological approaches to the measurement of HRQL may result in the identification of other relevant correlates [7], some of which are likely to vary among older men and women.

## Conclusion

As expected, we observed important decrements in clients' HRQL for common health conditions predictive or indicative of disability, including arthritis, mental health disorders and incontinence. For both females and males, HUI2 scores varied little with the presence of several other prevalent clinical diagnoses. Most of the observed variation in clients' HRQL scores was accounted for by two factors, poor self-rated health and caregiver distress. Although the direction of these associations are unclear, both factors may serve as important markers of psychosocial frailty and increased risk for poor client outcomes including future declines in HRQL. Despite sex differences in the prevalence of social and clinical factors likely to affect HRQL, most did not vary significantly by sex in their relative impact on HUI2 scores. However, our findings highlight several areas worthy of further investigation with larger samples, including possible sex differences in the relative importance of arthritis, incontinence, mental health and self-reported loneliness. Further demonstration of a differential impact of these factors among female and male clients may assist in the identification of appropriate sex-specific strategies for risk screening and care management of vulnerable seniors in community care settings.

## Competing interests

DHF has a proprietary interest in Health Utilities Incorporated, Dundas, Ontario, Canada. HUInc. distributes copyrighted Health Utilities Index (HUI) materials and provides methodological advice on the use of HUI. All other authors declare that they have no competing interests.

## Authors' contributions

CJM developed the initial study, supervised data collection, directed data analyses and wrote and edited the manuscript. JK conducted the primary analyses. JW assisted with study design and implementation, data preparation and analyses. JZ assisted with data preparation and analyses. DBH assisted with study development and implementation. DHF assisted with study design and implementation. WW co-led the development of the initial study, supervised data collection and assisted with data analyses. All authors made meaningful contributions during the editing of the manuscript and approved the final version.
